# The presence of secondary circulating prostate tumour cells determines the risk of biochemical relapse for patients with low- and intermediate-risk prostate cancer who are treated only with external radiotherapy

**DOI:** 10.3332/ecancer.2018.844

**Published:** 2018-06-20

**Authors:** Nigel P Murray, Socrates Aedo, Cynthia Fuentealba, Eduardo Reyes, Simone Minzer, Aníbal Salazar

**Affiliations:** 1 Carabineros Hospital of Chile, Ñuñoa, Santiago 7770199, Chile; 2 College of Medicine, Finis Terrae University, Providencia, Santiago 7501015, Chile; 3 College of Medicine, Diego Portales University, Manuel Rodríguez Sur 415, Santiago 8370179, Chile; 4 DIPRECA Hospital, Vital Apoquindo 1200, Las Condes, Santiago 7601003, Chile

**Keywords:** prostate cancer, radiotherapy, circulating prostate cells, biochemical relapse

## Abstract

**Introduction:**

The classification of patients with prostate cancer is used to determine treatments based on risk factors. The presence of secondary circulating prostate tumour cells (CPCs) detected in peripheral blood after a curative treatment has been associated with a worse prognosis. We present a prospective study of CPC detection post radiotherapy and the oncological results.

**Patients and methods:**

All of the patients classified as low and intermediate risk that were treated with radiotherapy were included. Three months after finishing treatment, an 8-ml blood sample was taken to detect CPCs. Mononuclear cells were obtained using gel centrifugation, and CPCs were identified using immunocytochemistry with anti-prostate-specific antigen. Patients were classified as low-risk CPC positive or negative and intermediate-risk CPC positive or negative. The biochemical relapse-free survival analysis was determined based on a follow-up of up to 15 years using the Kaplan–Meier and Cox regression models. Biochemical failure was defined according to the Pheonix II criteria.

**Results:**

Of 241 patients, 181 (75.1%) were classified as low risk and 60 (24.9%) as intermediate risk. Biochemical failure was observed in 27.1% (49/181) of the low-risk prostate cancer participants and in 53.3% (32/60) of intermediate-risk participants after 15 years of follow-up. 20.4% (37/181) of the low-risk cancer participants had detectable CPCs in comparison with 43.3% (26/60) of the intermediate-risk cancer participants (*p* < 0.001 overall risk 2.98, confidence interval (CI) 95% 1.59–5.56; relative risk 2.12, CI 95% 1.41–3.19). Positive CPC patients had a worse prognosis, and a shorter time period until biochemical relapse, regardless of risk group. The biochemical relapse-free survival curves show that intermediate-risk participants who were CPC negative had a higher survival rate and slower disease progression than those participants who were low risk but CPC positive.

**Conclusions:**

CPC detection is a risk factor for biochemical relapse and could be useful in identifying patients that will need additional treatment.

## Introduction

The use of prostate-specific antigen (PSA) as a screening method for prostate cancer has led to diagnosis at earlier stages of the disease, with the majority of the diagnosed men having the nonpalpable clinically localised disease [1, 2]. Even though the percentage of patients with tumours clinically confined to the prostate has increased, there are still treatment failures. External beam radiation therapy is one of the standard therapies for the treatment of localised prostate cancer.

Patients are classified into risk groups using pre-treatment PSA values, the Gleason score from the biopsy, and the clinical stage of the tumour [3]. This classification is used to define the treatment; low-risk patients can be treated with radiotherapy alone, while intermediate-risk patients are candidates for androgen deprivation therapy (ADT) after radiotherapy. Nonetheless, recent studies have questioned the benefit of ADT use for patients classified as intermediate risk [4].

It has been reported that by using three-dimensional conformal radiation therapy at doses greater than 76 Gy, that the biochemical relapse-free survival rates at 5 and 10 years are 94.1 and 79.2%, respectively for low-risk cancer patients, and 86.4 and 70.9%, respectively for intermediate-risk cancer patients [5]. Biochemical recurrence or relapse was defined according to the Phoenix criteria of ‘ASTRA II (American Society for Therapeutic Radiology and Oncology)’ as a PSA serum level of 2 ng/ml over the PSA nadir obtained after radiotherapy [6].

Risk classification is used to help make clinical decisions about patient management, providing a risk allocation or survival probability before treatment selection. The presence of secondary circulating prostate tumour cells (CPCs) detected in peripheral blood after a curative treatment has been associated with a worse prognosis for post-radical prostatectomy patients and implies the persistence of prostate cancer or minimal residual disease (MRD) [7].

We present this study that evaluates men with localised prostate cancer treated with 3D conformal radiation therapy alone as well as the presence or absence of CPCs detected 3 months after treatment and its association with therapeutic results, with up to 15 years of follow-up.

## Methods and patients

Male patients who underwent 3D conformal radiation therapy as their only therapy for prostate cancer between the years 2001 and 2016 were included.

Informed consent was obtained from each patient in order to record the following data: Age, date of surgery, diagnostic PSA using the Siemens Advia CentaurXR system, clinical status based on digital rectal exam according to the TNM classification system from 1997 [8], and Gleason score determined by a prostate biopsy analysed by a pathologist specialised in prostate cancer. In 2005, the Gleason score was modified according to the recommendations and consensus of the International Society of Urological Pathology [9], but because of the long time period of the study, the new score was not incorporated and the old system was maintained.

The patients were classified according to D’Amico as low, intermediate or high risk [3]. Only low- or intermediate-risk patients that did not receive ADT were included in the study. Men who had bone lesions in imaging studies were excluded, as well as patients who might have previously received ADT.

All of the patients received 3D conformal radiation therapy in daily fractions of 2 Gy, 5 days a week, without boosters, for an average dose of 75 Gy to the prostate (range: 74.1–76 Gy).

During the follow-up, the total serum PSA levels were measured every 3 months during the first 2 years and then every 6 months until biochemical recurrence or until the last check. Biochemical relapse was defined as an increase in the plasma levels of serum PSA of 2 ng/ml above the nadir obtained after radiotherapy according to the ‘Phoenix’ criteria of ASTRA II (American Society for Therapeutic Radiology and Oncology) [6].

## Detection of circulating prostate cells

Three months after completing radiotherapy, 8 ml of venous blood was taken and stored in a tube with ethylendiaminotetraacetic acid as an anticoagulant (BD Vacutainer, USA). The samples were stored at 4°C and were processed within the first 48 hours from collection. CPC detection was conducted by independent professionals who were blinded to the results of the other clinical pathologist.

In order to collect CPCs, mononuclear cells were separated by differential centrifugation, using Histopaque 1.077 (Sigma-Aldrich), they were washed and re-suspended in aliquots of 100 μL of autologous conditioned plasma. Aliquots of 25 μL were used in order to make the four sheets (sialinised, DAKO, USA) which were air-dried for 24 hours and set with a solution of 70% ethanol, 5% formaldehyde and 25% phosphate-buffered saline (PBS) at pH 7.4 for 5 minutes and finally washed three times in PBS at pH 7.4.

The CPCs were detected by way of immunocytochemistry using monoclonal antibodies against PSA, clone 28 A4 (Novocastro Laboratory, UK), and identified using an alkaline phosphatase anti-alkaline phosphatase reaction system (LSAB2, DAKO, USA) with new fuchsine as a chromogen. The positive samples were submitted to a second process with anti P504S clone 13H4 (DAKO, USA), and were identified with a system based on peroxidase (LSAB2, DAKO, USA) with DAB (3, 3 diaminobenzidine tetrahydrochloride) as a chromogen. A CPC was defined according to the criteria of International Society of Hemotherapy and Genetic Engineering [10] and the expression of P504S according to the consensus of the American Society of Pathologists [11]. A CPC was considered to be a cell that expresses PSA and P504S, since a benign prostate cell can express PSA but not P504S, and leukocytes can express P504S but not PSA. Patients with CPCs that were PSA positive but P504S negative were classified as negative for the test.

The samples were analysed manually, the dyed cells were photographed using a digital camera, and the presence or absence of CPCs and the total number of CPCs per sample were determined based on the digital images.

## Statistical analysis

The Stata program (Stata/SE 14.0 for Windows, Stata Corp Lp, 20159) was used, and the nature and distribution of quantitative and ordinal variables with central tendency measures (mean and median) and dispersion were described, using the interquartile range (IQR) and the standard deviation (SD) [12]. The Shapiro–Wilk test was used in order to define the null hypothesis regarding normal distribution. The nominal dichotomous variables were described as proportions with their respective confidence intervals [12].

Four groups were constructed based on the classification of risk group (low and intermediate) and the presence or absence of CPCs, Group 1: CPC negative and low risk; Group 2: CPC negative and intermediate risk; Group 3: CPC positive and low risk and Group 4: CPC positive and intermediate risk.

A nonparametric analysis of survival after 15 years of follow-up was conducted, establishing the survival percentage, median and mean for the entire cohort, as well as by established groups. In addition, a log-rank test was conducted comparing the survival rate among defined groups.

In the assessment of the presence or absence of CPCs and risk classification (low and intermediate), the biochemical failure prediction stops at 15 years.

The *hazard ratio* (HR) was determined for each pre-established group. The model’s validity was evaluated through the comparison between the predictions predicted by the model and those actually observed in the Kaplan–Meier curve. The model’s discriminating capacity and predictive accuracy is demonstrated by the assessment of Harrell’s C-index [12, 13].

The restricted mean survival time (RMST) is the average time in which an event occurs. Its clinical interpretation depends on the event studied and the follow-up time. It is determined by calculating the area under the survival curve [14, 15].

## Ethical considerations

This study was approved by the Local Ethics Committee and complies with the Declaration of Helsinki. All patients gave written consent.

## Results

The study included 241 men with organ-confined prostate cancer who met all of the inclusion criteria. Their ages were symmetrically distributed, with an average age of 67.0 ± 8.6 years old. At the time of diagnosis, serum PSA was asymmetrically distributed, with a median and IQR of 5.35 and 0.66 ng/mL, respectively.

Out of the 241 patients, 181 (75.1%) were classified as having low-risk organ-confined prostate cancer, and 60 (24.9%) as having intermediate risk. The clinical characteristics of the patients are given in Table 1. PSA and age contrasted with the Shapiro–Wilk test with a *P* value of <0.01. Out of the 241 patients, 65 (26.97%, confidence interval (CI) 95% 21.38–32.57) were CPC positive and 61 (25.31%, CI 95% 19.82–20.80) were intermediate risk. The distribution by the prognostic groups observed was 144 cases (59.75%, CI 95% 53.56–65.94) for group 1, 32 cases (13.28%, CI 95% 8.99–17.56) for group 2, 36 cases (14.94%, CI 95% 10.44–19.44) for group 3, and 29 cases (12.03%, CI 95% 7.93–16.14) for group 4.

For the study cohort at 15 years, the Kaplan–Meier survival curve for biochemical failure observed was 51.46% (CI 95%, 42.38–59.80), with no median observed. The restricted average for biochemical failure at 15 years of follow-up in all participating patients was 10.83 years (CI 95%, 10.14–11.53). Generally speaking, patients with a biochemical recurrence were older, with an average age of 69.5 ± 5.3 years old compared to 67.3 ± 3 years old (*P* < 0.001 *T*-test). They had a higher median serum PSA upon diagnosis, with a median of 5.89 ng/mL (IQR 2.4) compared to 5.21 ng/mL (IQR 1.60) (*P* < 0.001 Mann–Whitney test). They were also CPC positive in 72% of cases compared to 3% (*P* < 0.001 Mann–Whitney test).

**Figure 1.**
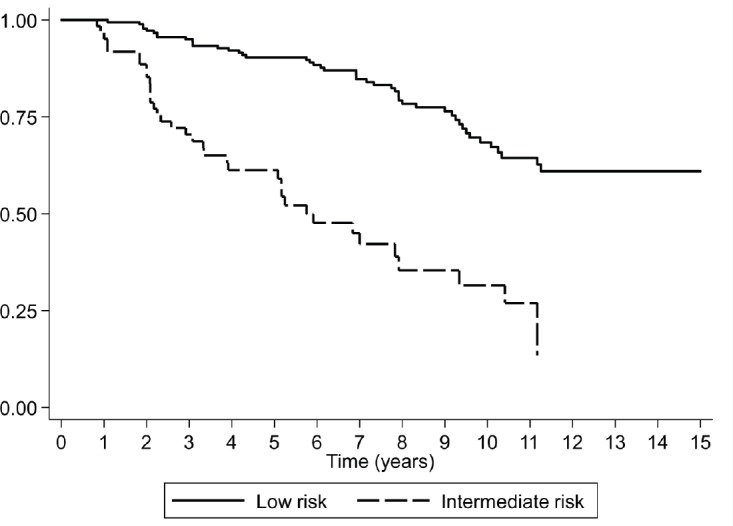
Survival rate observed (Kaplan–Meier), according to risk classification for a cohort of 241 subjects with prostate cancer followed over 15 years.

After 15 years of follow-up, biochemical recurrence was observed in 27.1% (49/181) of the patients with low-risk prostate cancer and in 53.3% (32/60) of those with intermediate risk. The Kaplan–Meier curves for men with low- and intermediate-risk prostate cancer are shown in Figure 1, demonstrating an HR of 4.13 (CI 95%, 2.61–6.53). The biochemical recurrence-free survival rate in low-risk prostate cancer at 5, 10 and 15 years was 90.3%, 68.4% and 65%, respectively. The median biochemical recurrence-free survival rate was not achieved in men with low-grade cancer. The RMST to biochemical recurrence was 12 years (CI 95%, 11.3–12.7 years) in low-risk patients.

For intermediate-risk prostate cancer patients, the biochemical recurrence-free survival rate at 5 and 10 years was 61.3 and 31.5%, respectively, with a median biochemical recurrence-free survival rate of 6 years, and a RMST to biochemical recurrence of 6.4 years (CI 95%, 5.3–7.4 years). Of the 60 intermediate-risk patients, 14 (23%) showed one risk factor, 35 (58%) showed two risk factors and 11 (19%) showed three risk factors. When evaluating for risk factors, 5/14 (36%), 21/35 (60%) and 9/11 (82%), respectively, had a biochemical recurrence.

### The presence or absence of CPCs and biochemical recurrence-free survival

20.4% (37/181) of the low-risk cancer participants had detectable CPCs in comparison with 43.3% (26/60) of the intermediate-risk cancer participants (*p* < 0.001 overall risk 2.98, CI 95% 1.59–5.56; relative risk 2.12, CI 95% 1.41–3.19).

**Figure 2.**
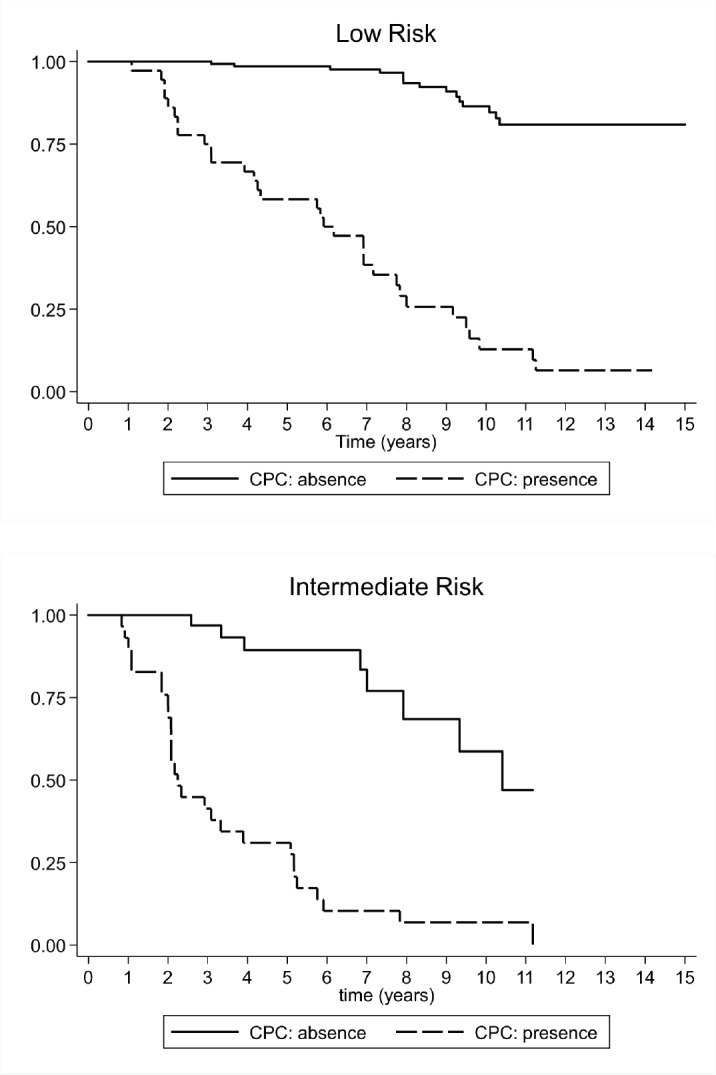
Survival rate observed (Kaplan–Meier), according to the presence or absence of CPC for 181 low-risk subjects, and 60 intermediate-risk subjects with prostate cancer followed over 15 years.

- Low-risk prostate cancer: CPC negative patients showed a higher biochemical recurrence-free survival rate after 15 years of follow-up. The Kaplan–Meier survival curve was 80.9% (CI 95%, 69.6–88.4%) at 15 years, the median survival was not achieved, and the RMST to biochemical recurrence was 13.8 years (CI 95%, 13.2–14.3 years). For CPC positive patients, the Kaplan–Meier survival rate at 15 years was 6.44% (CI 95%, 1.2–18.5 years), with a median biochemical recurrence-free survival of 5.9 years (CI 95%, 3.9–7.8 years), and a RMST to biochemical recurrence of 6.2 years (CI 95%, 5.1–7.4 years). The HR between both the groups was 16.7 (CI 95%, 8.5–32.8). However, in this group, the CPC negative patients demonstrated less treatment failure and the mean time to biochemical recurrence was significantly longer (Figure 2).
- Intermediate risk prostate cancer: CPC negative patients had longer biochemical recurrence-free survival rates at 15 years. The Kaplan–Meier survival rate was 47.0% (CI 95%, 17.6–72.0), with a median biochemical recurrence-free survival of 10.4 years (CI 95%, 7.9–12.0 years) and a RMST to biochemical recurrence of 9.3 years (CI 95%, 8.2–10.4 years). In turn, CPC positive patients had a Kaplan–Meier survival rate of 6.9% at 15 years (CI 95%, 1.2–19.8 years), with a median biochemical recurrence-free survival of 2.3 years (CI 95%, 2.0–3.6 years), and a RMST to biochemical recurrence of 3.6 years (CI 95%, 2.6–4.6 years). The HR between both the groups was 8.81 (CI 95%, 3.79–20.5). Just like in low-risk patients, the CPC negative patients in this group demonstrated less treatment failure and the mean time to biochemical recurrence was significantly longer (Figure 2).

### Biochemical recurrence-free survival risk based on CPC detection and risk group

Comparing the four groups in the study, there was a significant difference in the biochemical recurrence-free survival at 15 years, median survival and RMST to biochemical recurrence (Table 2). When comparing the biochemical failure-free survival between the prognostic groups, the log-rank test showed a *P* value of <0.01.

The predicted model for biochemical failure obtained through Cox proportional hazards regression shows an HR of 3.76 (CI 95%, 1.59–8.92) for CPC negative and intermediate-risk patients (group 2), an HR of 14.27 (CI 95%, 7.70–26.45) in CPC positive and low-risk patients (group 3) and an HR of 32.47 (CI 95%, 17.02–61.93) in CPC positive and high-risk patients (group 4). All of the aforementioned HRs show a *P* value of <0.05.

**Figure 3.**
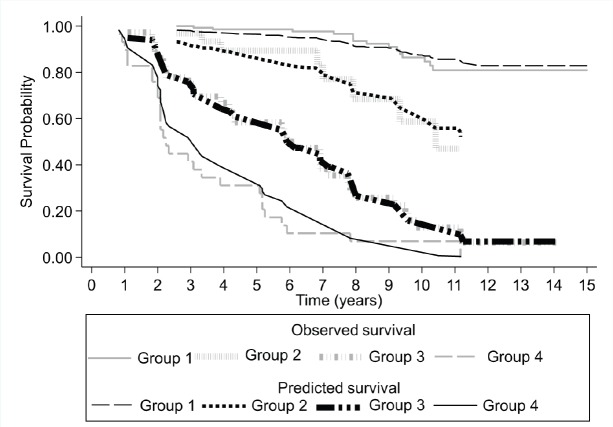
Survival rate observed (Kaplan–Meier), and predicted (Cox model), according to the presence or absence of CPC and group risk classification for a cohort of 241 subjects with prostate cancer followed over 15 years.

The survival described in the Kaplan–Meier parametric model (observed survival) is consistent with the survival predicted by the Cox proportional hazards regression model based on the presence of CPCs and the type of risk (low or intermediate) for biochemical failure (Figure 3), thus the predicted model shows adequate calibration.

Harrell’s C discrimination index for the predicted biochemical failure model based on the presence of CPCs and type of risk (low or intermediate) has a value of 0.85.

The biochemical recurrence-free survival curves show that intermediate-risk CPC negative patients had a higher survival rate and a longer time to disease progression than low risk but CPC positive patients (Figure 2).

## Discussion

It is important to consider identifying patients at risk of treatment failure after undergoing curative radiotherapy when managing prostate cancer patients. Patients classified as low risk have a very good prognosis after exclusive curative radiotherapy. In intermediate-risk patients, there is controversy as to the use of androgen blockade as an adjuvant therapy.

It has been suggested that intermediate-risk patients who show three risk factors, serum PSA at the time of diagnosis, Gleason score and clinical status should all receive androgen blockade [16]. However, the use of new biomarkers may help to classify high-risk patients and help in the management of such patients, especially patients who would not benefit from androgen suppression, and thus, would thus avoid adverse effects.

In our study group, the biochemical recurrence-free survival in low-risk patients was similar to that reported in the literature, whilst in intermediate-risk patients the recurrence-free survival was shorter. This can be explained by the number of patients with two or three risk factors, in whom the use of androgen blockade could have been considered.

CPC detection is dependent on the method used to detect them, and there is no consensus on which method is better. When using Cell-Search (Varidex, USA), a system based on epithelial cell adhesion molecule (EpCAM) and the only system approved by the Federal Drugs Administration, for the occurrence of CPC positive patients is less than 25% in patients with localised cancer and was similar to the control patients [17, 18]. However, by using a BER-EP4 antibody and a telomerase system, CPCs were detected in 80% of the organ-confined prostate cancer patients [19]. Methods using filtration to separate CPCs from peripheral blood have detected CPCs in all stages of prostate cancer [20, 21]. In this study, we have used gel centrifugation and immunocytochemistry to detect CPCs. Previously, in patients undergoing radical prostatectomy, we defined CPCs as primary when detected prior to surgery, and as secondary when detected after surgery. In order to differentiate between benign and malignant CPCs, we used dual immunolabelling. In patients with benign prostate diseases, it has been reported that cells that express PSA can be detected in the blood [22], but the detected CPCs do not express P504S, unlike malignant cells. In post-radiotherapy patients, it is possible that benign tissue that is inflamed by the radiation may result in benign CPCs spreading into the circulation. In patients with inflammatory colon diseases, cells expressing EpCAM and Cytokeratin were detected using the CellSearch method [23].

Monoclonal anti-PSA antibodies were used for their high specificity for prostate tissue. CPCs detected after radical prostatectomy have been associated with early biochemical recurrence [7]. We have internally validated this method of CPC detection using anti-PSA, with full agreement amongst different observers. It has the advantage of being of low cost, and it can be implemented in the routine laboratory testing of a general hospital. The results are reported as positive or negative to facilitate clinical decisions regarding patient management, and have been incorporated into local clinical guidelines. CPC negative patients have a follow-up according to the standard recommendations, whilst CPC positive patients have a more frequent follow-up. In the CPC positive group, possible treatments include observations, bisphosphonates and/or androgen suppression, as part of the clinical trial.

The first sample for CPC detection was taken 3 months post-treatment to minimise the inflammatory effect and to coincide with the first control with PSA, unlike post-prostatectomy patients where the first sample was 1 month post-treatment [24]. The CPCs detected post radiotherapy will be considered secondary CPCs, same as in post-radical prostatectomy patients. Their presence will not determine if they are from a local or distant microfocus, however, the progression-free survival of the disease is similar for CPC positive patients regardless of whether or not they have micrometastases in the bone marrow [25]. Therefore, in practice, CPC positive patients have a worse prognosis and a shorter time to recurrence than CPC negative patients [26].

For survival analysis, we used the restricted mean which is the mean time for an event to occur. Its clinical interpretation is dependent on the event under observation and the follow-up time. It is different from the median time in that it is a nonparametric test, which is the gold standard for a statistical test. It has been reported to have improved sensitivity in the analysis of time-dependent data [27].

In men with low-risk prostate cancer, 79.6% were CPC negative 3 months after radiotherapy. These patients had a very good prognosis, with more than 80% free of biochemical recurrence at 15 years post-treatment. The mean time to recurrence in these patients was close to 14 years, which suggests that androgen blockade would have had little benefit. However, men with low-risk cancer but that were CPC positive had a worse prognosis, with a biochemical recurrence-free survival of 6.4% at 15 years, and a mean time to recurrence of 6 years. These results suggest that it is possible to identify a subgroup of low-risk patients (20% of the total low-risk cancer group) who have a higher probability of treatment failure. It is of note that subjects in the intermediate risk group with negative CPCs had a much better prognosis, with a 47% 15-year recurrence-free survival rate, and a longer average time to recurrence of 9 years. However, men with intermediate-risk cancer with positive CPCs exhibited a worse prognosis, with a shorter average time to treatment failure of approximately 4 years, and an average survival rate free from biochemical recurrence of 2 years. Men with positive CPCs had a similar 15-year survival rate of approximately 6%; the difference in time to treatment failure was greater in low-risk men, six as opposed to 3 years.

In practical terms, the results of this study suggest the following.

- Patients with intermediate-risk prostate cancer with positive CPCs will be considered for early androgen blockade following the radiotherapy, as this group exhibited a low recurrence-free survival rate and a very short average time until failure. These findings suggest that these patients would benefit from early adjuvant therapy.
- Intermediate-risk patients with negative CPCs do not appear to benefit from early adjuvant therapy, since 50% were free of biochemical recurrence at 15 years, and those that exhibited curative radiotherapy failure had an average time until failure of 9 years. Observation of these patients is recommended until evidence of recurrence is presented.
- Low-risk prostate cancer patients with negative CPCs had an excellent prognosis, with more than 80% recurrence-free. These results suggest that these patients should be observed, without the need for adding additional therapy, as current guidelines recommend.
- Low-risk patients with positive CPCs had a worse prognosis than expected. Androgen blockade would be beneficial to these men, but this would require prospective clinical trials.

Our study has limitations, the first being that the Gleason grading system used was the old version, all patients were graded as Gleason ≤6, 7 or ≥ 8. The Gleason 3 + 4 and 4 + 3 grading recommended after 2005 [9] was not adapted. Whilst the concordance between the Gleason report on biopsy and the surgical specimen is greater [28] in terms of prognosis, there were no differences between the old Gleason 6 score and the new Gleason 3 + 4 score. In addition, comparisons between the historical and new results could be invalid [29–31]. For this reason, our study has kept the old Gleason grading system. Patients with Gleason 6 prostate cancer who meet the Epstein criteria [32] for prostate biopsy can be treated with active monitoring. It has been reported that the presence of CPCs prior to definitive treatment is linked to a more advanced cancer in terms of Gleason score and pathological state [33]. Whilst this has not been specified in this study, patients with Gleason 6 and treated with radiotherapy had a high rate of treatment failure when they were CPC positive. The suggestion is that not all Gleason 6 cancers are ‘benign’. This observation allows for the hypothesis that not all patients with Gleason 6 have low-risk cancer.

The use of immunocytochemistry through intra- and inter-observer variability has been questioned. However, the Kappa statistic for intra- and inter-observer variability for the presence or absence of CPCs was 0.77, which is considered good [24]. The clinical validity of a test is not necessarily the same as clinical utility. The results obtained in this study suggest that CPC detection has utility in the evaluation of patients treated with radiotherapy to predict the risk of biochemical recurrence. The results must be confirmed through multi-centric studies.

## Conclusions

The detection of CPCs post radiotherapy identifies a sub-group of patients at high risk of biochemical recurrence, independent of the low- and intermediate-risk classification. Nonetheless, the presence of CPCs as a biomarker for MRD does not define optimum treatment, as it does not distinguish between local or systemic recurrence.

The results merit random testing to confirm the utility of CPC detection in the prediction of biochemical recurrence in patients treated with radiotherapy.

## Conflicts of interest

Nigel Murray has received funding for consultations from Viatar CTC Solutions, USA. The other authors have reported no conflicts of interest.

## Funding

This study was supported by a research grant given by the Hospital de Carabineros in Chile.

## Figures and Tables

**Table 1.** Subject characteristics: 241 men with low- and intermediate-risk prostate cancer treated with radiotherapy.

	Low risk	Intermediate risk
Number of patients	181	60
Age Average +/− SD	68.1 +/− 5.9 years	68.0 +/− 5.9 years
Gleason score: ≤6 7	181 –	17 43
PSA ng/ml average pre-treatment IQR	5.90 4.68–6.06	6.96 5.25–11.69

**Table 2.** Survival rate, median survival rate, average survival rate restricted until biochemical failure and HR after 15 years follow-up, in accordance with low- and intermediate-risk prostate cancer, and the presence or absence of CPCs in a cohort of 241 prostate cancer patients treated with radiotherapy.

	Kaplan–Meier survival rate (CI 95%)	Median survival (CI 95%)	Restricted average (CI 95%)	Hazard ratio (CI 95%)
Group 1 CPC negative, low risk	86.40 (76.85–92.21)	Not observed	9.71 (9.54–98.89)	1
Group 2 CPC negative, intermediate risk	58.68 (28.83–79.58)	Not observed	8.67 (7.79–9.54)	3.83 (1.50–9.76)
Group 3 CPC positive, low risk	12.88 (4.16–26.69)	5.92 (3.92–7.75)	5.90 (4.92–6.88)	15.86 (8.09–31.08)
Group 4 CPC positive, intermediate risk	6.9 (1.22–19.75)	2.25 (2.00–3.89)	3.48 (2.58–4.39)	36.75 (18.30–73.79)
